# Nitrogen‐doped Carbon–CoO_*x*_ Nanohybrids: A Precious Metal Free Cathode that Exceeds 1.0 W cm^−2^ Peak Power and 100 h Life in Anion‐Exchange Membrane Fuel Cells

**DOI:** 10.1002/anie.201811099

**Published:** 2018-11-27

**Authors:** Xiong Peng, Travis J. Omasta, Emanuele Magliocca, Lianqin Wang, John R. Varcoe, William E. Mustain

**Affiliations:** ^1^ Department of Chemical Engineering University of South Carolina Columbia SC USA; ^2^ Department of Chemical and Biomolecular Engineering University of Connecticut Storrs CT USA; ^3^ Department of Chemistry University of Surrey Guildford Surrey UK

**Keywords:** anion-exchange membrane fuel cell, cobalt oxide, electrocatalysis, oxygen reduction reaction

## Abstract

Efficient and durable nonprecious metal electrocatalysts for the oxygen reduction (ORR) are highly desirable for several electrochemical devices, including anion exchange membrane fuel cells (AEMFCs). Here, a 2D planar electrocatalyst with CoO_*x*_ embedded in nitrogen‐doped graphitic carbon (N‐C‐CoO_*x*_) was created through the direct pyrolysis of a metal–organic complex with a NaCl template. The N‐C‐CoO_*x*_ catalyst showed high ORR activity, indicated by excellent half‐wave (0.84 V vs. RHE) and onset (1.01 V vs. RHE) potentials. This high intrinsic activity was also observed in operating AEMFCs where the kinetic current was 100 mA cm^−2^ at 0.85 V. When paired with a radiation‐grafted ETFE powder ionomer, the N‐C‐CoO_*x*_ AEMFC cathode was able to achieve extremely high peak power density (1.05 W cm^−2^) and mass transport limited current (3 A cm^−2^) for a precious metal free electrode. The N‐C‐CoO_*x*_ cathode also showed good stability over 100 hours of operation with a voltage decay of only 15 % at 600 mA cm^−2^ under H_2_/air (CO_2_‐free) reacting gas feeds. The N‐C‐CoO_*x*_ cathode catalyst was also paired with a very low loading PtRu/C anode catalyst, to create AEMFCs with a total PGM loading of only 0.10 mg_Pt‐Ru_ cm^−2^ capable of achieving 7.4 W mg^−1^_PGM_ as well as supporting a current of 0.7 A cm^−2^ at 0.6 *V* with H_2_/air (CO_2_ free)—creating a cell that was able to meet the 2019 U.S. Department of Energy initial performance target of 0.6 V at 0.6 A cm^−2^ under H_2_/air with a PGM loading <0.125 mg cm^−2^ with AEMFCs for the first time.

Polymer electrolyte membrane fuel cells have long been considered as the future power source for transportation systems and portable devices due to their environmental friendly operation and high energy conversion efficiency.^1^, ^2^ Proton exchange membrane fuel cells (PEMFCs) are currently widely accepted as the most promising alternatives to internal combustion engines, and PEMFCs are already being used to power thousands of fuel cell electric vehicles (FCEVs). However, the widespread application of PEMFCs has been limited by high costs, including the use of platinum group metal (PGM) electrocatalysts, which account for approximately one‐quarter of the cost of PEMFC‐based FCEV systems.^3^

In recent years, anion exchange membrane fuel cells (AEMFCs) have been highly touted as a possibly much lower cost electrochemical powerplant than PEMFCs. From a catalytic perspective, the alkaline environment means fundamentally enhanced kinetics for the oxygen reduction reaction (ORR) in AEMFCs compared to PEMFCs. This can allow for the use of ORR catalysts at the AEMFC cathode that are PGM‐free, like Ag,^4^ or even precious metal (PM)‐free and hence less costly. The alkaline AEMFC environment also widens the possible materials chemistries throughout the FCEV system, which can allow for the use of more affordable bipolar plates and less expensive membranes. In addition, many of the highest performing AEMFCs in the literature have operated at low cathode pressures, meaning that the air loop in the balance of plant could possibly be simplified.^3^

In recent years, a significant amount of research has been conducted and great progress has been made in the search for a PGM‐free (or preferably PM‐free) ORR electrocatalyst in both acid and alkaline media. Much of the work in this area has focused on nonprecious transition metal‐based materials or metal‐free nitrogen‐carbon catalysts for the ORR in alkaline media, including metal oxides,^5^ graphitic carbons^6^ and metal‐carbon composites.^7^, ^8^, ^9^ Among these, heteroatom‐doped carbon materials coupled with transition metals such as nickel, cobalt, iron and manganese have been widely accepted as the most promising candidates to replace Pt, with several catalysts showing very high ORR activity in ex‐situ rotating disk electrode (RDE) experiments. Unfortunately, to date, high ex‐situ activity has not been translated in the literature into high performance in operating AEMFCs. In fact, the best performing AEMFCs in the literature using a PM‐free cathode have been able to achieve a peak power density of only 0.20–0.70 W cm^−2^ and maximum achievable current density of less than 2.0 A cm^−2^.^6^, ^9^, ^10^ Accompanying these lower than desired performance metrics is the fact that the in‐cell stability of PM‐free catalysts generally remains unexplored.^6^, ^9^ Thus, PM‐free catalysts are currently not able to compete with PGM‐based catalysts in AEMFCs in terms of single cell performance (1.5–1.9 W cm^−2^) and durability (ca. 500 h).^11^, ^12^, ^13^, ^14^, ^15^ PM‐free cathode catalysts have also not been able to even come close to meeting the 2019 U.S. Department of Energy (DOE) targets for performance (>0.6 V at 600 mA cm^−2^ on H_2_/air; maximum pressure of 1.5 atm_a_) or enabling AEMFCs with the target total PGM loading of ≤0.125 mg_PGM_ cm^−2^.^16^ The ability to meet these DOE targets in an operating fuel cell is not only a function of the intrinsic activity of the catalyst, but also due to the accessibility of active sites during operation since mass transport is more complex in fuel cell catalyst layers (CLs) than liquid‐based thin film ex‐situ RDE experiments. Therefore, it is important to consider the final application in designing the chemistry and structure of new catalysts for fuel cell applications.

The ORR activity of PM‐free M‐N‐C (M‐Fe, Co, Mn, Ni, etc.) catalysts is generally thought to come from defect‐laden active sites^17^ where the transition metal (M), nitrogen (N) and carbon (C) coexist. The challenge with traditional structures, where the transition metal is supported by nitrogen doped carbon (N‐C), is that the catalysts tend to have very low active site density. This means that even though a catalyst active site may have a high turnover frequency, the volumetric reaction rate can be quite low. Low volumetric active site density translates directly into thick fuel cell CLs with notoriously poor mass transport properties, and, by extension, lower in‐cell performance than desired.^9^, ^10^ Therefore, to achieve PM‐free catalysts with high in‐cell AEMFC performance, it is necessary to develop electrocatalysts where their morphology may facilitate a higher density of M‐N‐C sites, and facilitate facile bulk mass transport.

Carbon shell embedded nanomaterials have recently attracted strong interest for energy conversion and storage applications.^18^, ^19^, ^20^, ^21^ In this design, a carbon shell is deposited onto the active material, providing physicochemical protection, electronic conductivity, as well as high interaction area between the active material and the carbon. This is exactly the family of properties that are expected to be advantageous for PM‐free AEMFC cathodes (as long as the inter‐particle pore structure can be controlled to allow for facile mass transport). Here, we report the synthesis and excellent properties of a well‐defined 2D, planar‐structured ORR electrocatalyst with cobalt oxide (CoO_*x*_) embedded into a casing of nitrogen‐doped graphitic carbon, denoted as N‐C‐CoO_*x*_. The N‐C‐CoO_*x*_ was fabricated via a facile, scalable heat treatment in a NaCl template, which simultaneously decomposed glucose, cobalt nitrate and ethylenediaminetetraacetic acid (EDTA) at 700 °C. The NaCl template was chosen because it provides a thermally stable supporting surface that is inexpensive and easy to recycle. Glucose was used as the carbon source because it is known to form graphitic carbon at relatively low temperature, below 750 °C,^19^ which helps to avoid significant loss of nitrogen from the EDTA during calcination. An illustration of the synthesis process is provided in Figure 1, and the synthesis details are provided in the Supporting Information.

**Figure 1:**
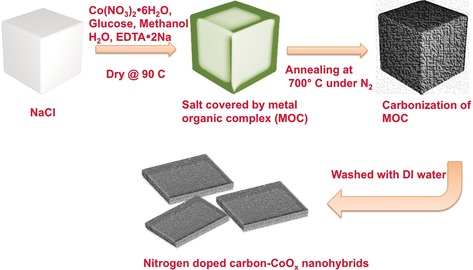
Schematic illustration of the synthesis procedure to create the nitrogen‐doped carbon CoO_x_ nanohybrids that resulting in very high performing AEMFC cathodes.

The resulting catalyst was comprised of 75 wt. % CoO_*x*_ (determined by thermogravimetric analysis, Figure S1 in the Supporting Information), 2 % nitrogen (determined by X‐ray photoelectron spectroscopy, XPS, Figure S2 and Table S1) and the balance carbon. The X‐ray diffraction pattern for N‐C‐CoO_*x*_ is shown in Figure S3. The broad peak observed at 23.5° can be attributed to the graphite (002) reflection from the carbon.^22^ The diffraction peaks at 31.32°, 36.64°, 38.84°, 44.86°, 59.22°, 65.28° and 69.54° can be assigned to the (220), (311), (222), (400), (511), (440) and (442) reflections for Co_3_O_4_ (JCPDS card no. 43‐1003), and the diffraction peaks at 61.62° and 77.60° can be assigned to the (220) and (222) reflections in CoO (JCPDS card no. 43‐1004), suggesting that a mixed phase oxide was created during synthesis. The morphology and microstructure of the N‐C‐CoO_*x*_ catalyst were probed through scanning electron microscopy (SEM), transmission electron microscopy (TEM) and scanning transmission electron microscopy (STEM). SEM images of N‐C‐CoO_*x*_ (Figure S4a,b,c) showed planar, thin (20–50 nm) carbon nanosheets with embedded CoO_*x*_ nanoparticles (Figure S4d). To show the importance of EDTA in forming the 2D nanostructure, the synthesis procedure was performed in the absence of EDTA, and though the resulting C‐CoO_*x*_ catalyst still exhibited an embedded nanoparticle morphology, the sheets were no longer planar (Figure S5).

The carbon nanosheet structure was further investigated by TEM. Lower resolution TEM images (Figure 2 a,b) confirmed the 2D planar structure with densely packed CoO_*x*_ particles. Higher resolution images (Figure 2 c) clearly showed that the CoO_*x*_ particles were surrounded by thin, onion‐like carbon layers within the 2D carbon nanosheets. Elemental mapping by energy dispersive X‐ray spectroscopy (EDS) showed that the Co, O, N and C elements were uniformly distributed over the sampled area (Figure 2 d–h). Overlaying the Co and C signals (Figure 2 i) also clearly showed that the CoO_*x*_ nanoparticles were confined in the N‐C matrix. This structure allows for the broad distribution and high density of reactive metal‐nitrogen moieties.

**Figure 2:**
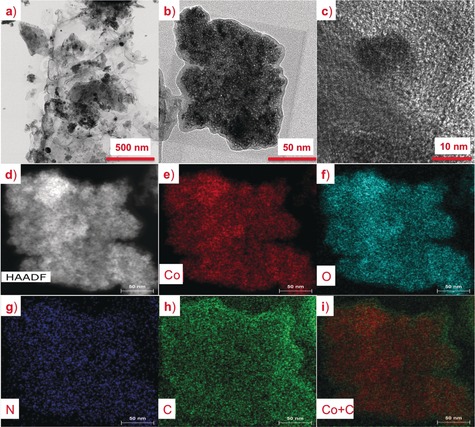
Morphology and elemental distribution of N‐C‐CoO_*x*_. a,b) Bright field TEM images at different magnification showing a 2D planar structure with densely packed CoO_*x*_ nanoparticles; c) HRTEM images showing CoO_*x*_ embedded in the N‐C matrix; d) HAADF‐STEM of the 2D N‐C‐CoO_*x*_ nanosheet; and corresponding e) Co, f) O, g) N, and h) C elemental maps; i) overlaid elemental maps for Co and C showing that the CoO_*x*_ nanoparticles were confined in the N‐C matrix.

The electrocatalytic activity of the N‐C‐CoO_*x*_ catalyst was evaluated by cyclic voltammetry (CV) and RDE measurements in O_2_‐saturated 0.1 m KOH solution, and compared to control materials that were: i) CoO_*x*_ embedded in N‐free carbon (C‐CoO_*x*_); ii) raw CoO_*x*_ without any encapsulation (CoO_*x*_); iii) sulfuric acid treated N‐C‐CoO_*x*_ (leaving elemental Co at the N‐C sites, but removing bulk CoO_*x*_), denoted as SA‐Co‐N‐C; and iv) N‐C prepared without any CoO_*x*_. As shown in Figure 3, all three components of the N‐C‐CoO_*x*_ catalyst was necessary to achieve high activity. Increased activity was shown not only by the more positive position of the ORR cathodic peaks in the CV in Figure 3 a,^7^ but by more positive half wave and onset potentials in the RDE voltammograms (Figure 3 b). In the RDE environment, CoO_*x*_ showed the lowest ORR activity, though after the CoO_*x*_ was coated with carbon (C‐CoO_*x*_) the charge transfer resistance was significantly decreased (Figure S7), which resulted in considerably improved activity.

**Figure 3:**
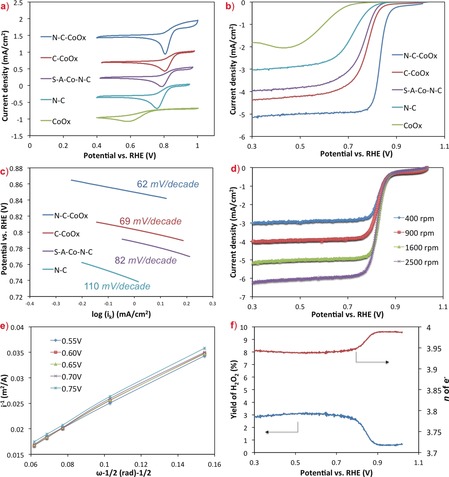
a) Cyclic voltammograms for N‐C‐CoO_x_, C‐CoO_x_, CoO_x_, SA‐Co‐N‐C and N‐C in 0.1 m O_2_‐saturated KOH electrolyte at scan rate of 10 mV s^−1^ at room temperature; b) ORR polarization curves for N‐C‐CoO_*x*_, C‐CoO_*x*_, CoO_*x*_, SA‐Co‐N‐C, N‐C in 0.1 m O_2_‐saturated KOH at scan rate of 5 mV s^−1^ at 1600 rpm; c) Tafel plots for N‐C‐CoO_*x*_, C‐CoO_*x*_, SA‐Co‐N‐C and N‐C, determined by mass‐transport correction of the 1600 rpm RDE data; d) ORR polarization curves for N‐C‐CoO_*x*_ at different rotation rates; e) Koutecky‐Levich (*KL*) plots for the ORR on N‐C‐CoO_*x*_ at different potentials; f) Number of electrons (*n*) transferred per O_2_ molecule and hydrogen peroxide yield on the N‐C‐CoO_*x*_ catalyst as a function of potential.

After the inclusion of N in the carbon matrix, the N‐C‐CoO_*x*_ catalyst showed the highest activity—with the highest half‐wave (0.84 V vs. RHE) and onset (1.01 V vs. RHE, Figure 3 b) potentials—and the lowest Tafel slope (62 mV dec^−1^, Figure 3 c)—very promising results. Even when compared to commercial Pt/C (BASF, 50 %), the highest performing ORR catalyst in AEMFCs, the N‐C‐CoO_*x*_ performed very well. In fact, the N‐C‐CoO_*x*_ half‐wave potential was only ≈20 mV more negative than Pt/C (Figure S8), making N‐C‐CoO_*x*_ one of the most active PGM‐free electrocatalysts reported in the literature to date.^9^, ^23^, ^24^, ^25^

The ORR mechanism on N‐C‐CoO_*x*_ was further investigated by rotating ring disk electrode (RRDE) voltammetry at several rotation rates between 400–2500 rpm (Figure 3 d) and performing a Koutecky‐Levich analysis.^26^ The Koutecky‐Levich plots from the RRDE disk are shown in Figure 3 e, and the peroxide yield from the ring is shown in Figure 3 f. The average number of electrons transferred (*n*) was 3.9 and the HO_2_^−^ yield was stable at ca. 3 % over the entire potential window of interest (0.30–0.80 V vs. RHE). Thus, the overwhelmingly dominant ORR mechanism on the N‐C‐CoO_*x*_ catalyst is the four‐electron (4e^−^) reduction of O_2_, with the first electron transfer being the rate‐determining step. The combination of high activity, low production of unwanted peroxide, and its high surface area and open structure make N‐C‐CoO_*x*_ an ideal candidate material for the AEMFC cathode.

Next, the N‐C‐CoO_*x*_ catalysts were mixed with ETFE solid powder ionomers,^27^ dispersed in solvent and sprayed onto gas diffusion layers to create PM‐free gas diffusion electrodes (GDEs).^14^ SEM images of the GDEs (Figure S9a,b) showed a uniform distribution of catalyst and ionomer particles as well as a very porous architecture, which is highly beneficial to *operando* reactant and product mass transfer.^28^ Energy‐dispersive X‐ray spectroscopy (Figure S9d) clearly showed that the ionomer was well integrated with the catalyst (Figure S9c), which suggests that these electrodes are likely to have a well‐formed triple‐phase boundary in operating AEMFCs.

The cathode GDEs were used to construct lab‐scale, single‐cell AEMFCs that could be used to test the in situ N‐C‐CoO_*x*_ activity and stability under realistic operating conditions. The membrane used in this work was low‐density polyethylene (LDPE) (25 μm, IEC=2.87±0.05 mmol g^−1^) with covalently‐bound benzyltrimethylammonium (BTMA) cationic head‐groups.^11^ First, the operating AEMFCs were fed with pure H_2_ and O_2_ reacting gases, where a few important observations were made. First, the N‐C‐CoO_*x*_ catalyst was able to achieve very high kinetic current in the operating AEMFC, 100 mA cm^−2^ at 0.85 V. This in‐cell kinetic behavior compares extremely well to the existing state‐of‐the‐art in both AEMFCs^6^, ^9^, ^29^ (Figure S10a) and PEMFCs^30^, ^31^ (Figure S10c), even though many of the previous works were done at higher temperature (particularly PEMFC). Second, the N‐C‐CoO_*x*_ cathode was able to achieve a mass transport limited current density of 3 A cm^−2^ and a maximum power density of 1.05 W cm^−2^—both of which are the highest reported values for a PM‐free cathode in AEMFCs to date (Table S3, Figure S10b).^6^, ^9^, ^10^, ^15^, ^29^, ^32^, ^33^, ^34^, ^35^, ^36^ Such high power density and achievable current density shows that the reported catalyst, integrated with the ionomer, enabled the creation of catalyst layers that: i) have much lower mass transport resistance than previous PM‐free cathodes in operating AEMFCs; and ii) are competitive with PM‐free PEMFC cathodes. Third, by surpassing the 1.0 W cm^−2^ threshold, this is the first PM‐free electrode that can compete with state‐of‐the‐art Pt/C cathodes in operating AEMFCs.

To further explore its feasibility for commercial use, the behavior of the N‐C‐CoO_*x*_ GDE was evaluated under several additional conditions. First, air (CO_2_‐free) was used as the oxidant, and the N‐C‐CoO_*x*_ GDE was able to support a mass transport limited current of 2.5 A cm^−2^ and achieve a peak power density of 0.66 W cm^−2^. Second, the total PGM loading of the MEA was reduced to 0.10 mg_PtRu_ cm^−2^ (which is below the DOE target of 0.125 mg_PGM_ cm^−2^) by coupling the N‐C‐CoO_*x*_ GDE with a thin PtRu/C anode GDE.^32^ This very low PGM‐loading cell was able to support high peak power density of 0.73 W cm^−2^ under H_2_/O_2_ reacting gases, equating to a remarkable specific power output of 7.4 W mg^−1^_PGM_—the highest of any AEMFC to date (Figure S11).

Despite the high performance, an obvious limiting factor in this cell was water management, which is gaining attention as a critical consideration for AEMFC performance and durability,^15^, ^28^ which is indicated by the small difference in performance under O_2_ and air feeds as well as the curvature of the polarization curve at high current densities. Finally, the N‐C‐CoO_*x*_ GDEs were subjected to short‐term stability testing under both H_2_/air (Figure 4 c) at 600 mA cm^−2^ and H_2_/O_2_ (Figure S12) at 300 mA cm^−2^. Under H_2_/air reacting gas flows, the cell showed promising short‐term stability, with a small voltage loss (ca. 15 %) over the 100 h experiment. A polarization curve was collected after the 100 h test, which also showed a voltage loss of around 15 % at 600 mA cm^−2^ (Figure S13). After the durability test, the most significant change was in the mass transport regime where the mass transport limited current was surprisingly reduced by 40 %, which will have to be investigated further in future work. Operating under H_2_/O_2_ reacting gas flows, the cell also did not see significant degradation over 70 hours, though adequate water management was unfortunately not achieved, indicated by spikes in the cell voltage that are most likely due to the accumulation and quick release of water within the low loading, hence thin, anode.

**Figure 4:**
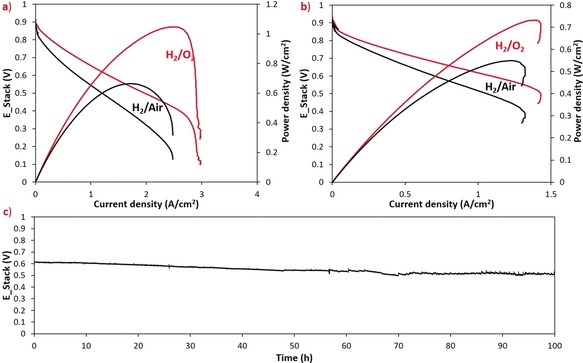
AEMFC performance and stability with N‐C‐CoO_*x*_ cathodes. a) Polarization curves and power densities as a function of current density for H_2_‐O_2_ and H_2_‐air (CO_2_‐free) fuel cells; cathode: 2.4 mg cm^−2^ of N‐C‐CoO_*x*_, 0.08 MPa backpressure; anode: 0.70 mg cm^−2^ of PtRu, 0.12 MPa backpressure; *T*_cell_=65 °C (data presented without *iR*‐correction). Polarization curves and power densities for H_2_‐O_2_ and H_2_‐air (CO_2_‐free) fuel cells with a very low PGM‐loading anode; cathode: 2.4 mg cm^−2^ of N‐C‐CoO_*x*_, 0.09 MPa backpressure; anode: 0.10 mg cm^−2^ of PtRu, 0.1 MPa backpressure; *T*_cell_=65 °C (data presented without *iR*‐correction). c) Stability of H_2_/air (CO_2_‐free) AEMFC operating at 600 mA cm^−2^; cathode: 2.4 mg cm^−2^ of N‐C‐CoO_*x*_, 0.2 MPa backpressure; anode: 0.70 mg cm^−2^ of PtRu, 0.2 MPa backpressure (data presented with *iR*‐correction). The membrane in this work was a LDPE‐BTMA AEM (IEC=2.87±0.05 mmol g^−1^)^11^ and the ionomer was ETFE‐BTMA powder.^27^

After AEMFC stability testing, the cell was disassembled and N‐C‐CoO_*x*_ was collected by abrasive removal from the CL and subjected to TEM to examine the evolution in its morphology during testing and to evaluate possible degradation mechanisms. As shown in Figure S14, an overwhelming majority of the N‐C‐CoO_*x*_ was able to preserve its 2D nanosheet structure (Figure S14 a, b) with embedded CoO_*x*_ (Figure S14 c, d), providing evidence for excellent operando stability for this cathode. There is also no obvious inter‐particle agglomeration between CoO_*x*_ particles. However, a small amount of cobalt oxide dissolution was observed, which resulted in the formation of void spaces in the N‐containing carbon. There was also evidence of cobalt re‐deposition (and re‐oxidization) in weak affiliation with the N‐C (Figure S15 a–c). However, it should be noted that the N‐C was very stable (Figure S14 d–f), and an overwhelming majority of the CoO_*x*_ remained embedded in the N‐C matrix. In combination, the ex‐situ RDE and operando AEMFC performance and stability of the N‐C‐CoO_*x*_ catalyst represents a promising new pathway to creating high performing PM‐free catalysts for AEMFCs and other electrochemical devices.

In conclusion, a facile, low cost and scalable method was used to fabricate a highly active and stable N‐C‐CoO_*x*_ catalyst, which was comprised of CoO_*x*_ nanoparticles confined in 2D nitrogen‐doped carbon nanosheets. The N‐C‐CoO_*x*_ had very high ex situ ORR activity, which was translated into an operating AEMFC where the catalyst was able to achieve a high in situ activity of 100 mA cm^−2^ at cell voltage of 0.85 V. Additionally, when combined with the ionomer in operating AEMFCs, the N‐C‐CoOx cathode was able to support a peak power density of 1.05 W cm^−2^—unprecedented for a precious‐metal(PM)‐free AEMFC electrode. AEMFCs were also prepared where the N‐C‐CoO_*x*_ cathode was paired with a very low PGM loading anode, 0.10 mg cm^−2^ PtRu, and the cells were able to achieve a new record for specific power: 7.4 W mg^−1^_PGM_. The N‐C‐CoO_*x*_ catalyst also showed very promising stability over 100 h of operation. These results not only significantly narrow the gap between high performing PGM‐containing and PM‐free ORR cathodes, but also point to the promise of AEMFCs as a low‐cost alternative to PEMFCs for both stationary and mobile applications as well as provide a new direction for the design of ORR catalysts in alkaline media.

## Conflict of interest

The authors declare no conflict of interest.

## Supporting information

As a service to our authors and readers, this journal provides supporting information supplied by the authors. Such materials are peer reviewed and may be re‐organized for online delivery, but are not copy‐edited or typeset. Technical support issues arising from supporting information (other than missing files) should be addressed to the authors.

Supplementary
