# Supplementary material for: Nitrogen‐doped Carbon–CoOx Nanohybrids: A Precious Metal Free Cathode that Exceeds 1.0 W cm−2 Peak Power and 100 h Life in Anion‐Exchange Membrane Fuel Cells
Source: Angew Chem Int Ed Engl. 2018 Nov 27;58(4):1046–51. doi: 10.1002/anie.201811099 (PMC6468319; doi:10.1002/anie.201811099)
Supplement: Supplementary file 1 — Supplementary [file ANIE-58-1046-s001.pdf]

Angewandte Chemie
Eine Zeitschrift der Gesellschaft Deutscher Chemiker

Supporting Information

Nitrogen-doped Carbon–CoO_x Nanohybrids: A Precious Metal Free Cathode that Exceeds 1.0 $W\,cm^{-2}$ Peak Power and 100 h Life in Anion-Exchange Membrane Fuel Cells

*Xiong Peng, Travis J. Omasta, Emanuele Magliocca, Lianqin Wang, John R. Varcoe, and William E. Mustain**

anie_201811099_sm_miscellaneous_information.pdf

1. Experimental

1.1. Electrocatalyst sysnthesis

Synthesis of N-C-CoO_x As illustrated in Figure 1 of the primary manuscript: 0.439 g of $Co(NO_3)_2$-$6H_2O$, 0.25 g of glucose, 80 mg of EDTA-2Na and 15 g of NaCl were dissolved in mixed solvent of methanol (10 mL) and H_2O (10 mL) in a 80 mL beaker. The mixture was heated at 90 °C with string rate of 600 rpm until it was entirely dry, during which, the formed Co-EDTA-glucose complex was coated on surface of NaCl crystal[1]. After that, the mixture was ground in a mortar to very fine powders followed by direct calcination at 700° C under N_2 with ramp rate of 6 °C/min for 3 h. As a result, the metal organic complex on NaCl surface was converted to nitrogen-doped carbon CoO_x nanohybrids (N-C-CoO_x) with uniform thickness of graphitic carbon nanosheets. This strategy enabled the protection of active sites by embedding CoO_x into graphitic carbon framework to prevent interparticle agglomeration and meal dissolution during fuel cell operation. Besides, the graphitic carbon nanosheets would help provide higher electronic conductivity compared to pure metal oxides catalyst when applied in CL. The calcinated mixture was washed with mixed solvent of DI water and methanol (4:1) to remove NaCl and precipitated by centrifugation at 4500 rpm. Finally, the obtained power was dried at 60 °C before physical characterization and electrochemical measurements.

Synthesis of C-CoO_x. The C-CoO_x sample was synthesized using the same procedure as N-C-CoO_x without adding EDTA•2Na.

Synthesis of SA-Co-N-C. The N-C-CoO_x-acid treated sample was prepared by soaking N-C-CoO_x sample in aqueous 1M H_2SO_4 for 24 h and then washed with DI water and dried at 60 °C before electrochemical measurements.

Synthesis of CoO_x. The CoO_x was synthesized using the same procedure as N-C-CoO_x without adding EDTA•2Na and glucose.

Synthesis of N-C. The N-C was synthesized using the same procedure as N-C-CoO_x without adding metal precursor.

1.2. Material characterization and analysis

X-ray diffraction (XRD) patterns were obtained using Rigku MiniFlex II equipped with a high sensitivity D/tex Ultra Si slit detector. Data was collected from 20 to 90° at a scan rate of 0.0285°/s with a step size of 0.02° and a CuKα radiation source (λ = 0.1540562 nm) operated at 30 mA and 15 kV. The N-C-CoO_x as well GDEs surface morphology were imaged with a Zeiss Ultra plus field emission scanning electron microscope (FESEM). Transmission electron microscope (TEM) and scanning transmission electron microscope images were acquired on a FEI Talos S/TEM. The metal oxide content was determined by thermal gravimetric analysis (TGA) with a NETZSCH STA 449. The sample was firstly baked at 120 °C under N_2 to remove moisture and then heated in air at ramp rate of 10 °C/min to 1000 °C with a subsequent hold at this temperature for 30 min.

X-ray photoelectron spectroscopy (XPS) measurements were performed using a Kratos AXIS Ultra DLD XPS system with a monochromatic AlKα source operated at 15 keV and 150 W, and a hemispherical energy analyzer. The X-rays were incident at an angle of 45° with respect to the surface normal. The analysis was performed at a pressure below 10^{-9} mbar. Pass energy of 40 eV was employed to analyze high-resolution core-level spectra were measured with, and analysis of the data was carried out using XPSPEAK41 software. The high-resolution Co 2P spectra (Figure S6a) could be deconvoluted into two doublets with 57 % of Co^{3+} and 43 % of Co^{2+}, indicating a mixed oxidation state of Co,[2,3] which corresponds well with the XRD pattern (Figure S2). The chemical bonding states of N (Figure S6b) are comprised of pyridinic N (398.3 eV), graphitic N (400.6 eV) and N-oxide (404.2 eV), with dominate pyridinic and graphitic N speices (28.6 % and 62.2 %, respectively), which were believed to be favorable for electrocatalysis of ORR[4]. The high-resolution C1s and O1s spectra shows that the amount of carbon-oxygen species are up to 11.7 % (Figure S6c, S6d, Table S2), which have been shown to be important for ORR in alkaline media due to the formation of defects within graphene network[5].

1.3. Electrochemical measurements

Electrochemical measurements were conducted on a thin film rotating disk electrode (RDE) in a custom three-electrode cell (Adams & Chittenden Scientific Glass) using a platinum mesh as the counter electrode and a double junction Ag/AgCl reference electrode (Pine Research Instrumentation, 4 M aqueous KCL internal solution). Cyclic voltammograms and ORR polarization curves were recorded using an Autolab PGSTA302N potentiostat. The working electrode was prepared on a glassy carbon disk electrode (geometric area: 0.1962 cm^2; Pine Research Instrumentation) by dropping 18.5 µL of a catalyst ink with the following composition onto the electrode: 11 mg of non-PGM catalyst, 7.6 mL of IPA, 2.4 mL of DI water, and 50 µL of 5 % Nafion ionomer dispersion (DuPont). As for Pt/C (BASF, 50%) control, The working electrode was prepared on a glassy carbon disk electrode by dropping 13 µL of a catalyst ink with the following composition onto the electrode: 7.6 mg catalyst, 7.6 mL IPA, 2.4 mL DI water and 20 µL 5% Nafion ionomer dispersion (DuPont). The film was dried on a leveled, inverted rotator at 700 rpm at room temperature. Prior to thin-film deposition, the glassy carbon electrodes were polished with a 0.05 mm alumina suspension and carefully washed with 18.2 MΩ cm Millipore ultrapre water (UPW) and dried in air for 20 min. The catalyst loading on the glassy carbon were approximately kept at 0.11 mg/cm^2. The three-electrode cell was washed using UPW water and aqueous 0.1 M KOH (10 and 5 × before use, respectively).

The electron transfer number (n) is calculated with Koutecky-Levich equations:

$$\frac{1}{j} = \frac{1}{j_L} + \frac{1}{j_K} = \frac{1}{B\omega^{1/2}} + \frac{1}{j_K}$$

$$B = 0.62nFC_o(D_o)^{2/3}\nu^{-1/6}$$

where j is the measured current density, j_K is the kinetic current density, j_L is the mass transport limiting current density, ω is the angular velocity of the disk, F is Faraday Constant (96485C/mol), C_o is the bulk concentration of O_2 in 0.1 M KOH at room temperature (1.2×10^{-6} mol/cm^3), D_o is the diffusion coefficient of O_2 in 0.1 M KOH at room temperature (1.9×10^{-5} cm^2/s), υ is the kinematic viscosity for 0.1 M KOH (0.01 cm^2/s).

Rotating ring-disk electrode (RRDE) measurements were conducted in the same RDE setup as mentioned above. The disk electrode was rotated at 1600 rpm with scan rate of 10 mV/s. The ring electrode potential was set to 1.1 V vs. RHE. The hydrogen peroxide yield (%H_2O_2) and electron transfer number (n) were calculated by the following equations:

$$\%H_2O_2 = 200 * \frac{i_r/_N}{i_d + i_r/_N}$$

$$n = 4 * \frac{i_d}{i_d + i_r/_N}$$

where i_d and i_r are the disk and ring current densities, N is the ring H_2O_2 collection efficiency, which is 37 %.

1.4. GDE fabrication and AEMFC testing

Prior to formulation of the catalyst ink, an ETFE-g-poly(VBTMAC) powder anionomer (IEC = 1.24 ± 0.06 mmol/g)[6] was first ground with a well-cleaned mortar and pestle for 10 min to reduce the number of aggregated particles. Next, the catalyst and 1 mL of UPW water was added to the ground anionomer and ground for an additional 10 min until a visually homogeneous catalyst slurry was formed. The ETFE powder mass comprised of 20 % of the total solid mass of all of the catalyst layers (CLs) in this paper. After the slurry was homogenized, 1.5 mL of isopropanol was added into the mortar followed by another 5 min grinding. A final 5 mL of isopropanol was added to the mortar and the final ink mixture was transferred to a PTFE- lined vial and sonicated for 1 hour in ice bath. The prepared ink was then sprayed onto the GDL (Toray 60, 5% PTFE) using an air-assisted sprayer (Iwata) to fabricate GDEs. A Pt-Ru catalyst (Alfa Aesar HiSPEC 10000, Pt nominally 40%wt., and Ru, nominally 20%wt., supported on Vulcan XC-72R carbon) was used at the anode. Both the anode, cathode GDEs and membrane were hydrated in DI water for 20 min and then soaked three times in aqueous 1.0 M KOH to remove impurities and ion exchange the quaternary ammonium hydroxide groups before cell assembly.

AEMFCs with 5 cm^2 active area were assembled in single cell hardware with a single channel serpentine flow field. The Anion Exchange Membranes (AEMs) used in this work were a radiation-grafted low-density polyethylene (LDPE) film (25 µm, IEC=2.87 ± 0.05 mmol g^{-1}) with a covalently-bound benzyltrimethylammonium (BTMA) cationic head-groups.[7] The average thickness of anode and cathode were measured to be 216 and 270 µm, respectively. Thus, 152 and 203 µm Teflon gaskets were used on anode and cathode, respectively to keep the pinch around 25 % of the total GDE thickness. The AEMFCs were tested on a Scribner 850e fuel cell test station at a cell temperature of 60 °C under H_2/O_2 or H_2/Air flow at 1.0 L/min. The cell was pre-operated at a voltage of 0.5 V for break-in and the relative humidity (RH) of both the cathode and anode was adjusted to help the cell to be operated at optimal conditions.

1.5. Low PGM AEMFC electrodes fabrication.

In our case, the cathodes were the N-C-CoO_x electrodes. Anodes were fabricated using a different procedure from normal loading anodes. This low PGM loading anode was firstly sprayed with a layer of micro porous layer (MPL) with composition of Vulcan carbon and ETFE ionomer (ionomer:Vulcan = 0.417:1 wt. ratio) to avoid electrocatalyst

penetrated into GDL and enhance anode water capacity. The carbon loading in MPL layer was kept around 0.20 mg/cm^2. After that, a catalyst ink (comprised of 20 mg of PtRu/C, 100 mg of Vulcan carbon and 45 mg of ETFE ionomer) was sprayed onto MPL. The PGM loading was calculated by mass difference before and after spraying.

2. Figures and Tables

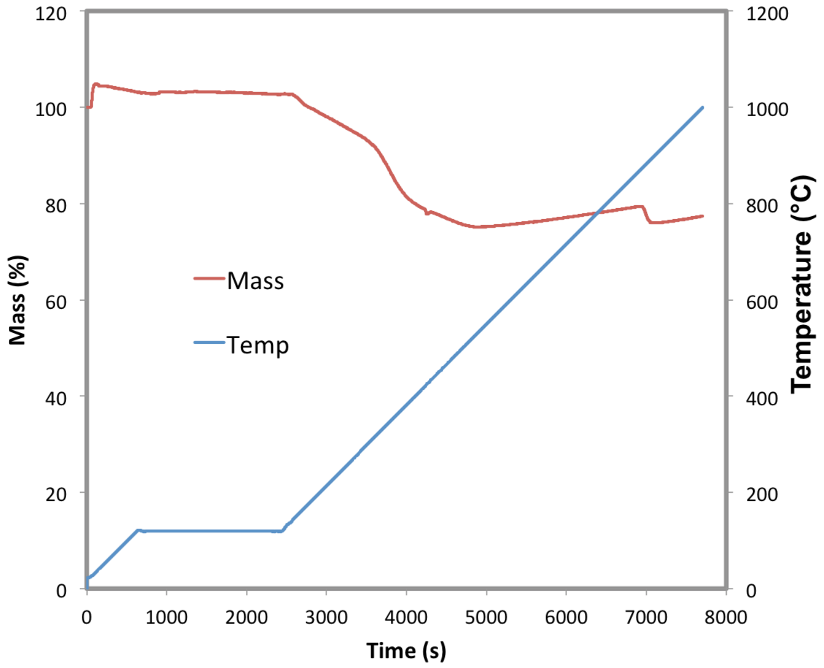

Figure S1. TGA profile of the N-C-CoO_x showing the metal content around 75 %.

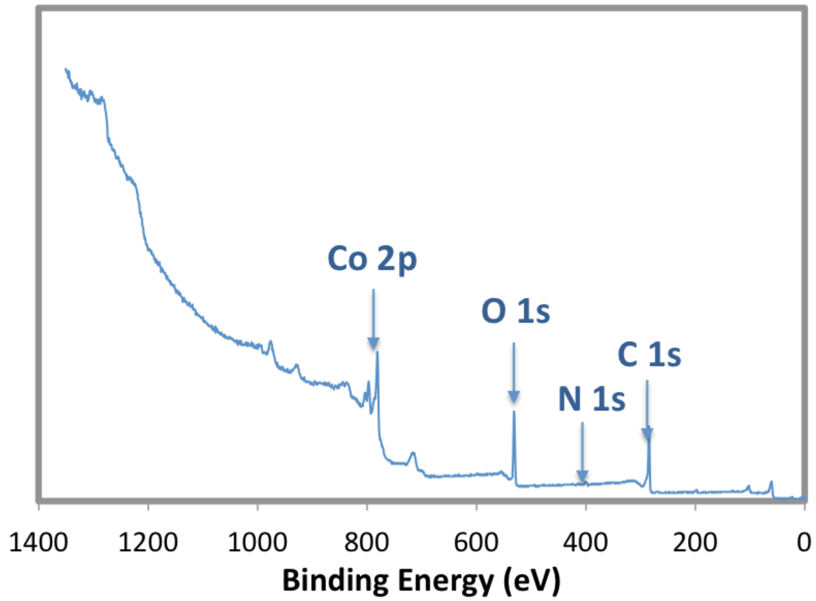

Figure S2. XPS survey scan of the N-C-CoO_x

Table S1. Element quantification of the surface of the C-C-O_x and N-C-CoO_x.

	C	N	O	Co
Mass %	37.3	1.4	17.2	44.1
Atomic %	61.7	2.0	21.4	14.9

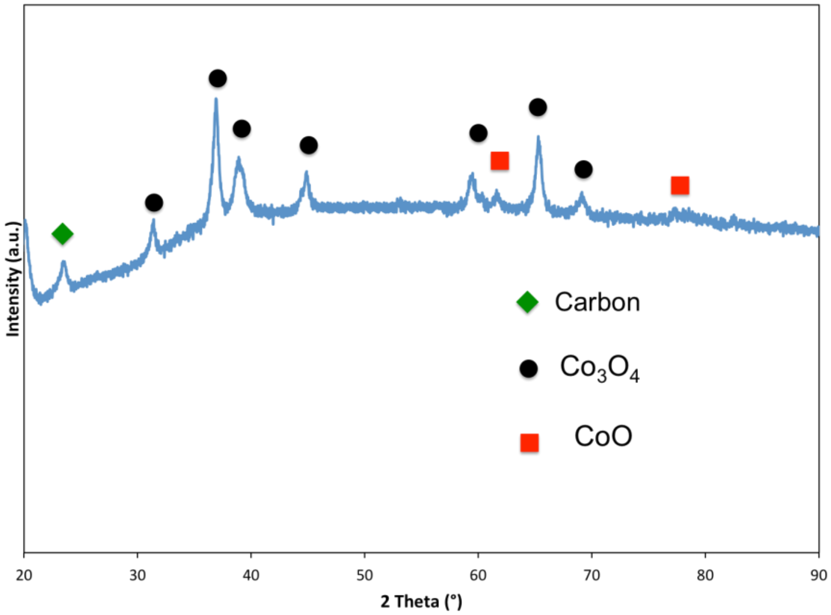

Figure S3. XRD pattern of the N-C-CoO_x.

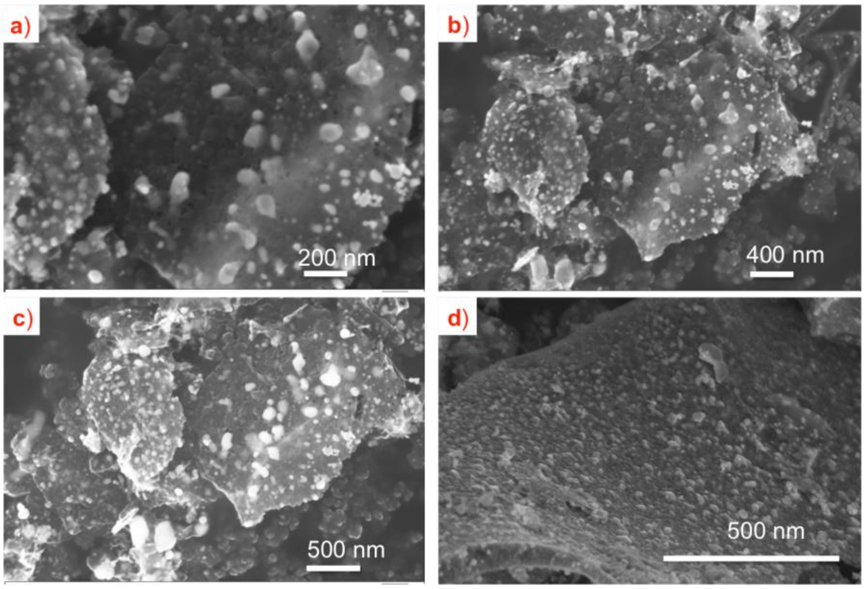

Figure S4. SEM images of the synthesized N-C-CoO_x.

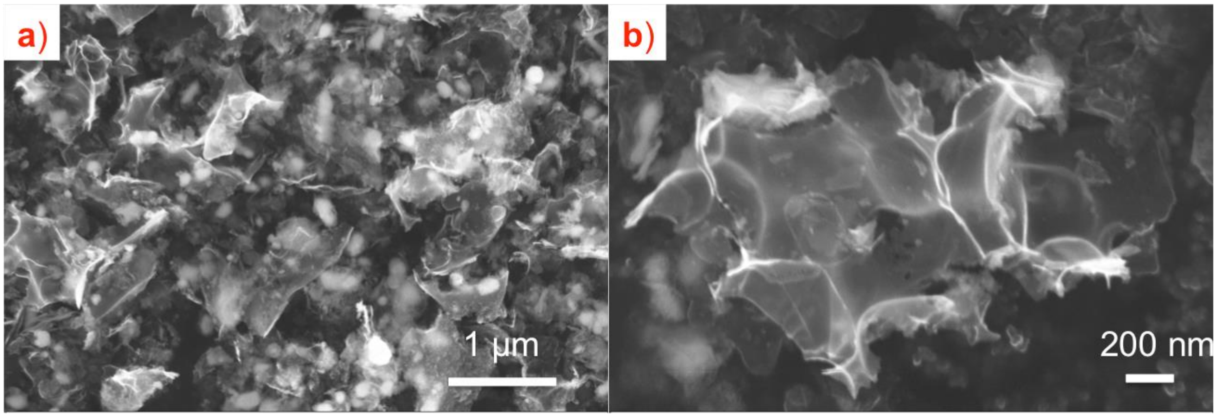

Figure S5. SEM images of the synthesized C-CoO_x.

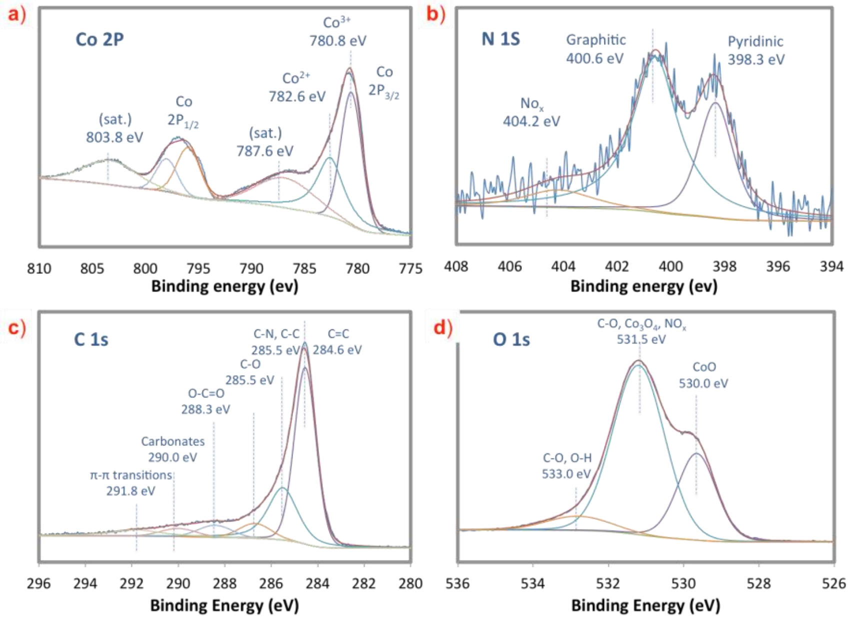

Figure S6. High resolution XPS of Co, N, C and O in N-C-CoO_x.

Table S2. The (%) ratio of each contribution in comparison to the total intensity of the C 1s peak is shown below.

	C=C	C-N, C-C	C-O	O-C=O	Carbonates	$\pi - \pi^*$
% ratio	53.4	26.2	6.3	5.4	3.8	4.9
Full Width at Half Maximum (FWHM)	1.3	1.5	1.6	1.7	1.8	2.5

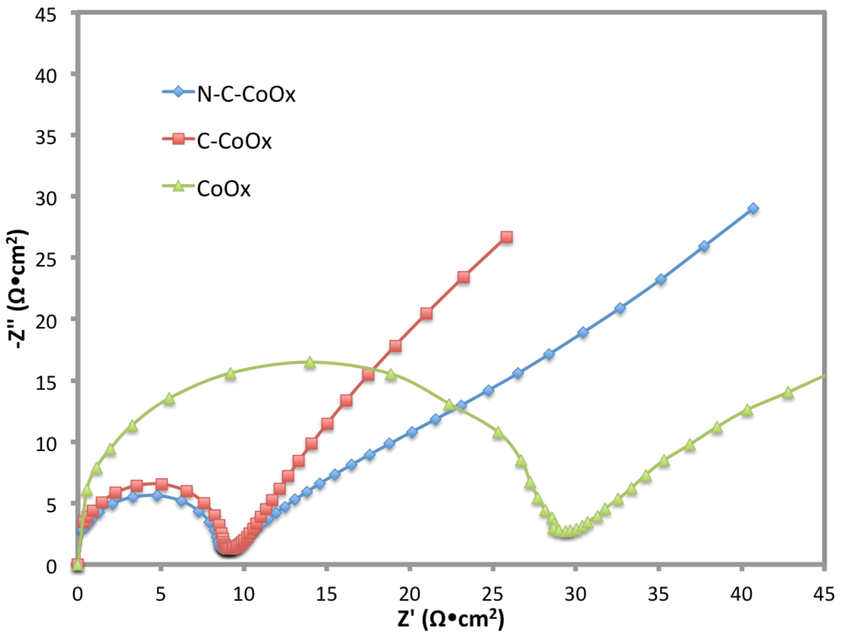

Figure S7. Nyquist plots of the N-C-CoOx, C-CoOx and CoOx. EIS frequency range was 10 kHz to 0.1 Hz. Data were taken in O_2-purged aqueous 0.1 M KOH under open circuit voltage (data presented with iR-correction).

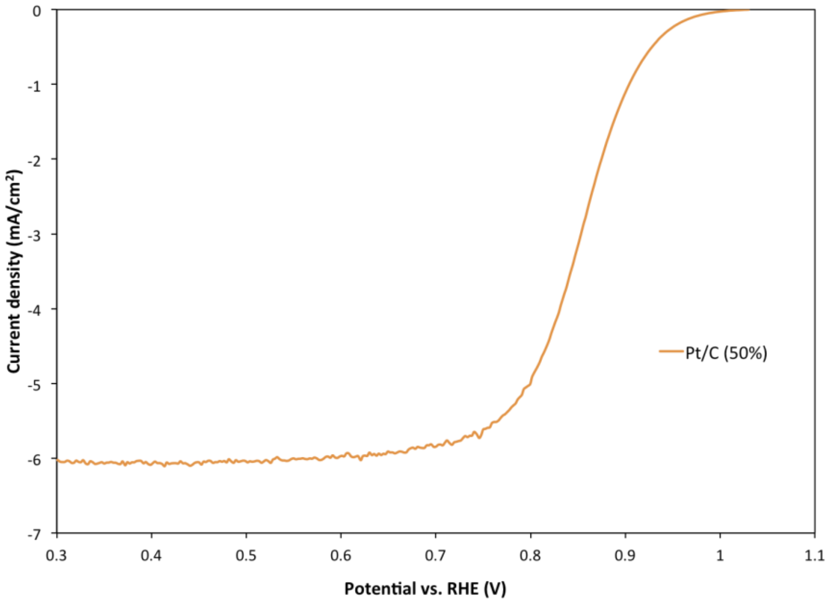

Figure S8. ORR polarization curves for Pt/C (50% BASF) in 0.1 M O_2-saturated KOH at scan rate of 5 mV s^{-1} at 1600 rpm. The half-wave potential was measured at 0.86 V, which was 20 mV higher than the N-C-CoO_x.

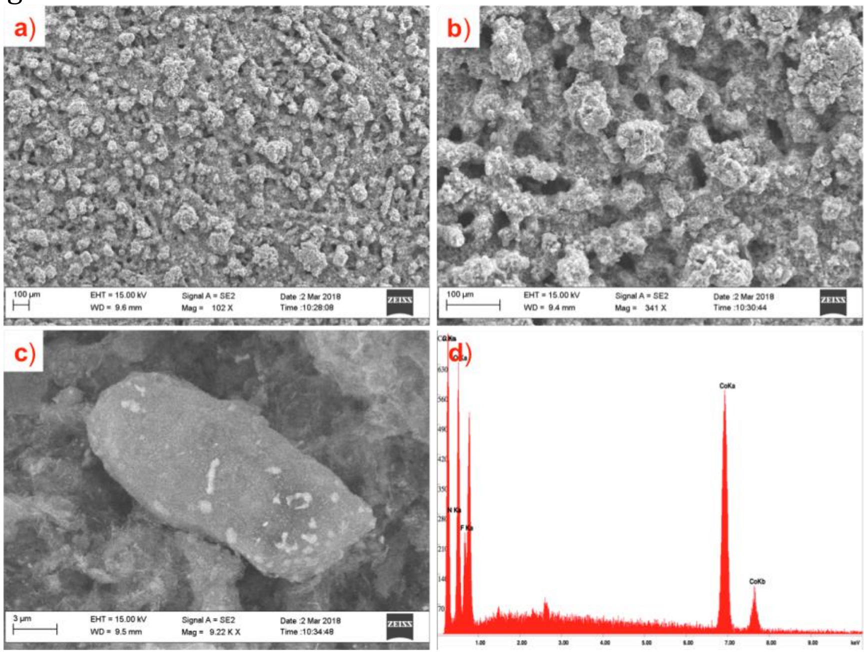

Figure S9. (a), (b) SEM images of the cathode GDE with N-C-CoOx catalyst under different magnifications; (c) SEM images showing the ETFE ionomer was wrapped by the catalyst, (d) the corresponding EDS showing the existence of C, N, F and Co element.

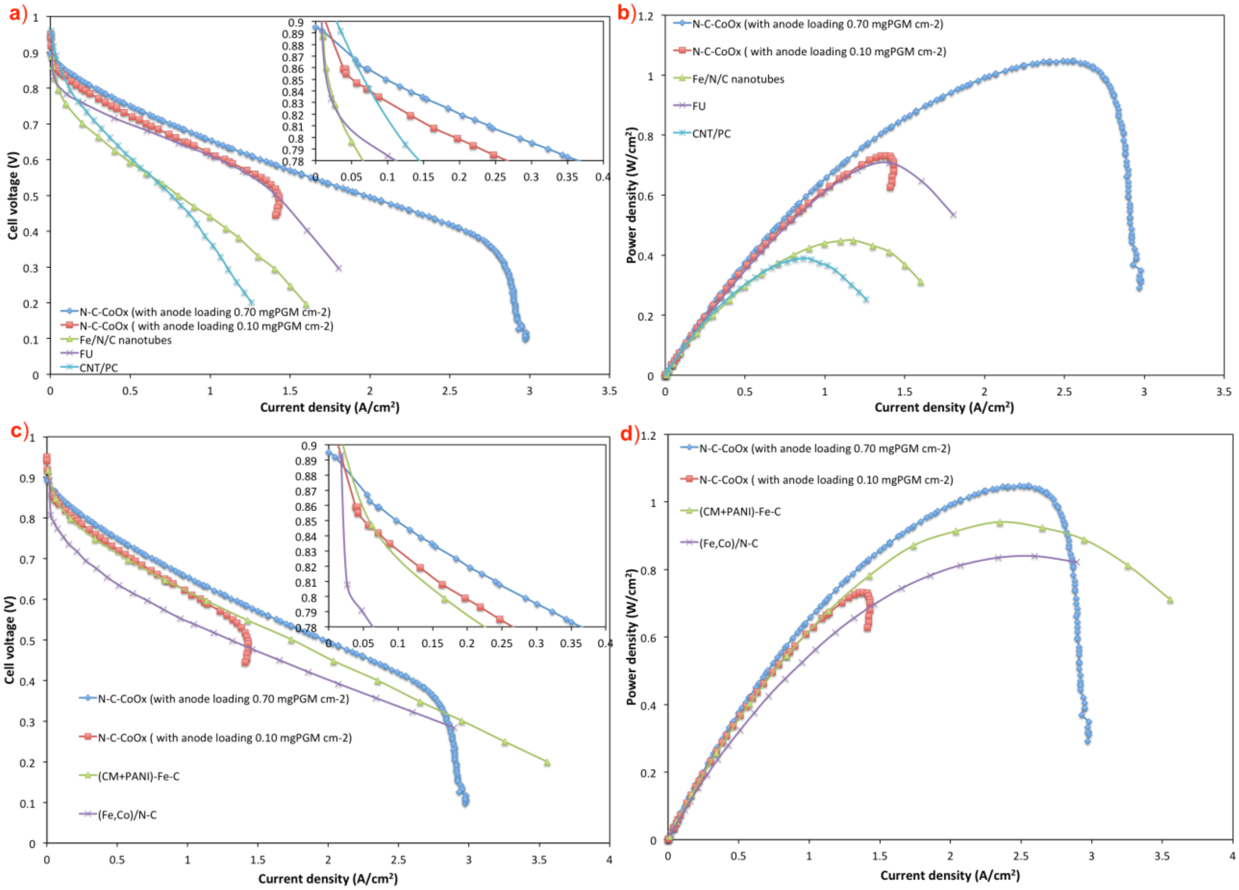

Figure S10. Comparison of single cell performance and kinetic region (inset) between this work and state of the art non-PGM cathode fuel cell work. a), b) i-V curves and i-power density curves between this work and non-PM cathode FU[8], Fe/N/C nanotubes[9] and CNT/PC[10] in AEMFC; c), d) i-V curves and i-power density curves between this work and (CM+PANI)-Fe-C[11], (Fe,Co)/N-C[12] in PEMFC. The result indicated that the N-C-CoO_x still exhibited better kinetic region and mass transport region than all of the non-PGM cathodes compared in AEMFC. Even compared to state of the art non-PGM cathodes in PEMFC using Nafion as membrane and electrolyte. The N-C-CoO_x also showed superiority in the kinetic region and comparably mass transport region.

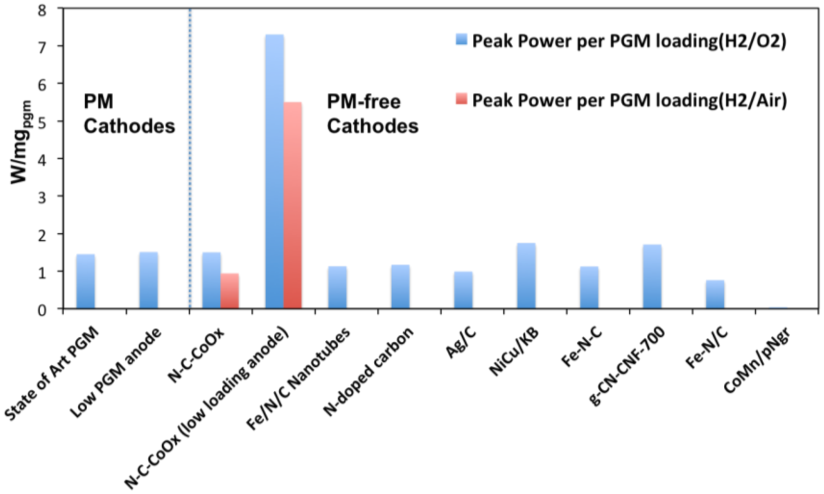

Figure S11. Comparison of specific peak power (W/PGM loading) between different AEMFCs: both state of the art PGM-electrodes and those employing non-PGM electrocatalysts[2-9].

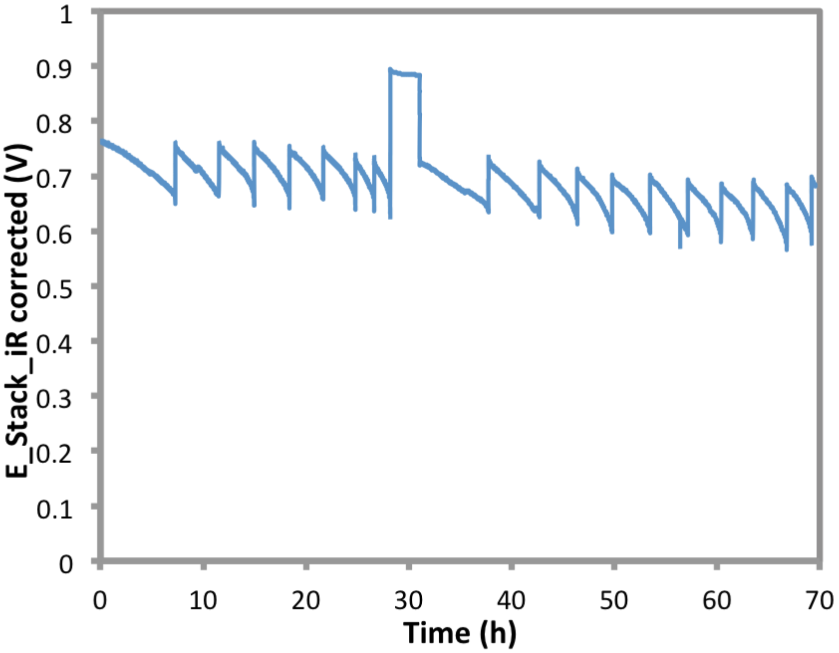

Figure S12. Stability testing at 300 mA/cm^2 under H_2/O_2 with Cathode: 2.4 mg/cm^2 of N-C-CoO_x; 0.09 MPa backpressure; anode: 0.10 mg/cm^2 of PtRu; 0.1 MPa backpressure. Due to fact that this anode had a PGM loadng of only 0.10 mg_{PtRu}/cm^{-2}, the electrode was more sensitive to flooding than high loading anodes (0.70 mg_{PtRu}/cm^{-2}). The bouncing of cell voltage between 0.65 to 0.75 V was most likely due to anode flooding and quick release of flooding. After 70 h running, the cell experienced less than 10 % peak-to-peak performance loss, which can also be considered as very good stability.

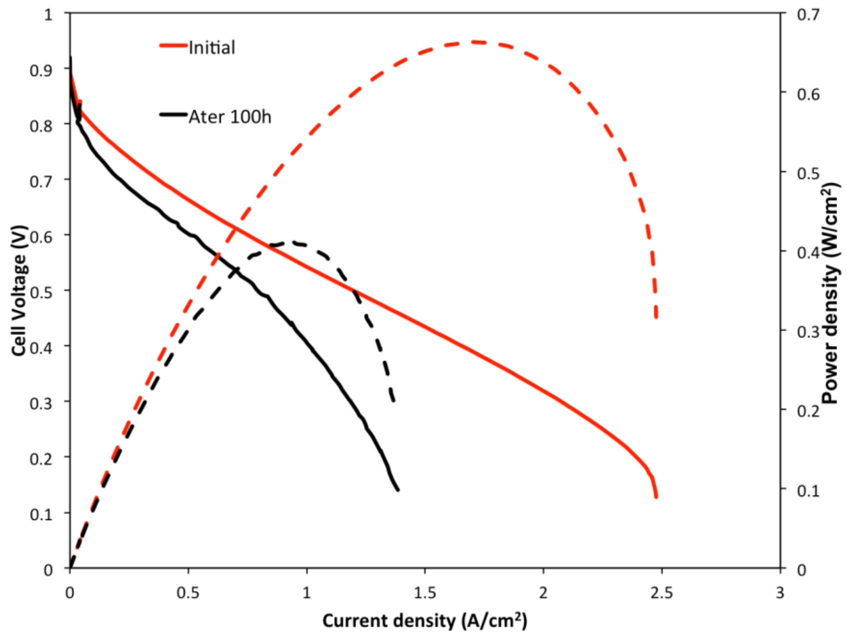

Figure S13. Comparison of polarization curves (solid lines) and power density (dashed lines) before and after 100h stability test under H_2/Air. cathode: 2.4 mg cm^{-2} of N-C-CoO_x, 0.2 MPa backpressure; anode: 0.70 mg cm^{-2} of PtRu, 0.2 MPa backpressure.

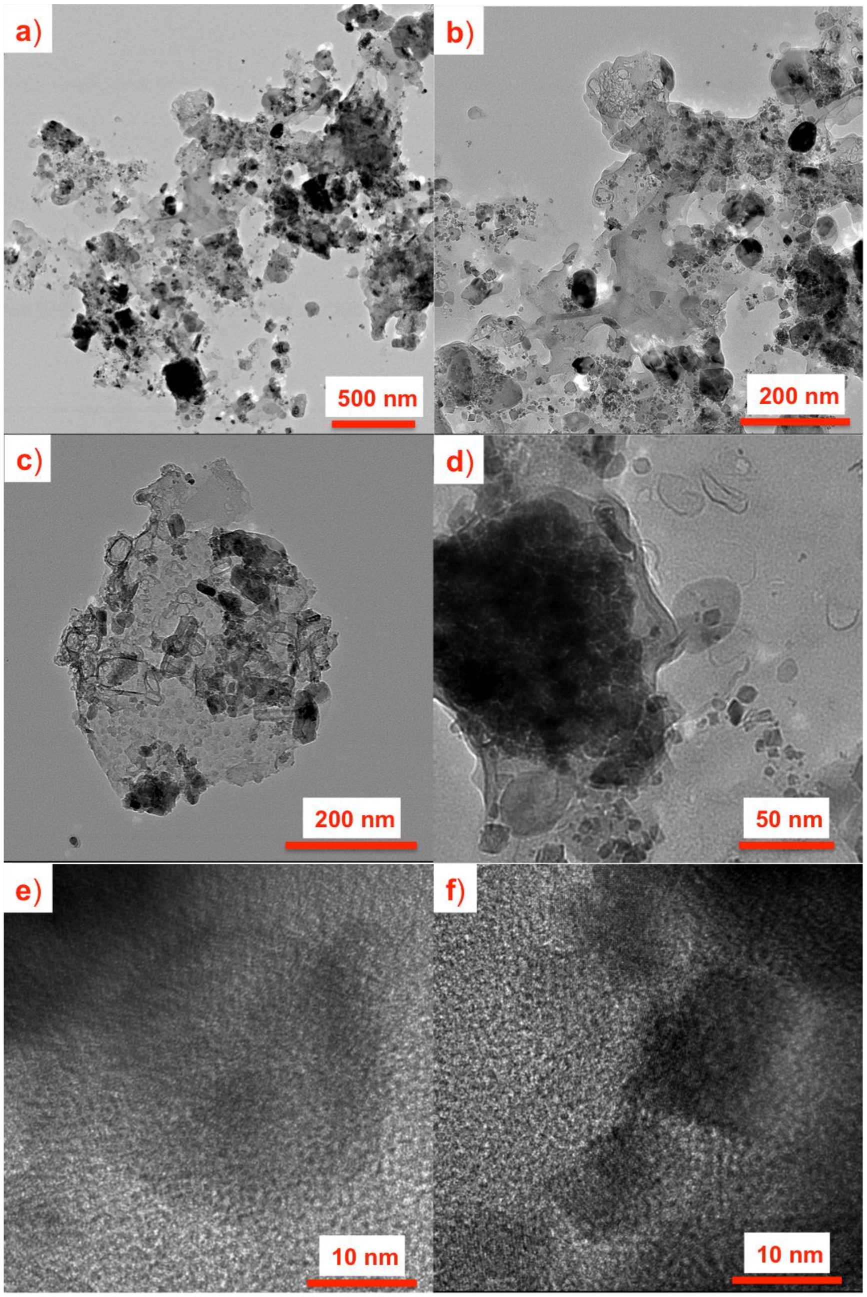

Figure S14. Morphology of N-C-CoO_x after stability testing: a), b), c) and d) Bright field TEM images at different magnifications; e), f) HRTEM images showing CoO_x embedded in carbon nanosheet.

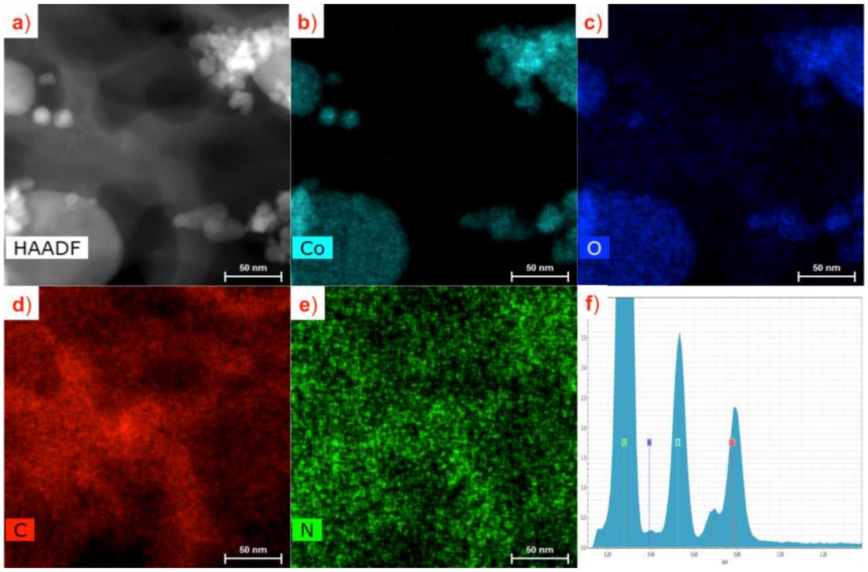

Figure S15. a) HAADF of N-C-CoO_x after stability testing; b), c), d) e) correspond to the Co, O, C and N mapping; (f) EDX spectra of the imaged area.

Table S3. AEMFC performance comparison with one side using non-PGM electrocatalysts and state of art PGM work.

Electrocatalyst	Catalyst loading (mg/cm^2)		Ionomer	Membrane	Current density (A/cm^2) at 0.8 V	Peak power density (W/cm^2)	Peak Power per PGM loading (W/mg_{PGM})	Operating condition			
	Cathode	Anode (PGM)						Back Pressure (Mpa) (Ca/An)	T_{cell} (°C)	RH (Ca/An)	Gas type (Ca/An)
N-C-CoO_x (This work)	2.4	0.70 (PGM)	ETFE	LDPE-BTMA	0.29 (O_2) 0.08 (Air)	1.05 (O_2) 0.66 (Air)	1.5 (O_2) 0.94 (Air)	0.08/0.12	65		H_2/O_2 H_2/Air
N-C-CoO_x (This work)	2.4	0.10 (PGM)	ETFE	LDPE-BTMA	0.25 (O_2) 0.11 (Air)	0.73 (O_2) 0.55 (Air)	7.3 (O_2) 5.5 (Air)	0.1/0.1	65		H_2/O_2 H_2/Air
Fe/N/C Nanotubes[9]	2.0	0.40 (PGM)	αQAPS-S_{14}	αQAPS-S_x	0.1	0.45	1.13	0.1/0.1	60	100/100	H_2/O_2
N-doped carbon[8]	1.0	0.6 (PGM)	ETFE	ETFE-BTMA	0.05	0.7	1.17	0/0	60	83/83	H_2/O_2
Ag/C[13]	1.0	0.2 (PGM)	AS-4	Tokuyama A201	0.1	0.198	0.99	0.2/0.2	80	100/100	H_2/O_2
NiCu/KB[14] (Anode)	0.2 (PGM)	4.0	AS-4	Tokuyama A201	0.125	0.35	1.75	0.14/0.14	80	100/100	H_2/O_2
Fe-N-C[4]	3.5	0.2 (PGM)	AS-4	Tokuyama A201	0.08	0.225	1.125	0.14/0.14	70	100/100	H_2/O_2
g-CN-CNF-700[15]	2	0.1 (PGM)	AS-4	Tokuyama A201	0.08	0.171	1.71	0.05/0.05	50	100/100	H_2/O_2
Fe-N/C[10]	2.0	0.5 (PGM)	VTLC	VTLC-PET	0.2	0.38	0.76	_______	80	100/100	H_2/O_2
CoMn/pNGr[16]	0.8	0.8	Fumion	FAA-3	0.02	0.03	0.0375	_______	50	100/100	H_2/O_2
State of Art PGM[17]	0.60	0.71	ETFE	ETFE-BTMA	0.8	1.9	1.45	0	60	58/60	H_2/O_2
Low PGM Anode[18]	0.11	0.52	ETFE	ETFE-BTMA	0.45	0.9	1.5	0	50		H_2/O_2

References:

[1] B. KARAMI, S. NIKOSERESHT, S. KHODABAKHSHI, *Chinese J. Catal.* **2012**, *33*, 298–301.

[2] D. Yu, Y. Wang, L. Zhang, Z. X. Low, X. Zhang, F. Chen, Y. Feng, H. Wang, *Nano Energy* **2014**, *10*, 153–162.

[3] J. Cui, X. Zhang, L. Tong, J. Luo, Y. Wang, Y. Zhang, K. Xie, Y. Wu, *J. Mater. Chem. A* **2015**, *3*, 10425–10431.

[4] M. M. Hossen, K. Artyushkova, P. Atanassov, A. Serov, *J. Power Sources* **2018**, *375*, 214–221.

[5] R. Gokhale, Y. Chen, A. Serov, K. Artyushkova, P. Atanassov, *Electrochem. commun.* **2016**, *72*, 140–143.

[6] S. D. Poynton, R. C. T. Slade, T. J. Omasta, W. E. Mustain, R. Escudero-Cid, P. Ocón, J. R. Varcoe, *J. Mater. Chem. A* **2014**, *2*, 5124–5130.

[7] L. Wang, J. J. Brink, Y. Liu, A. M. Herring, J. Ponce-González, D. K. Whelligan, J. R. Varcoe, *Energy Environ. Sci.* **2017**, *10*, 2154–2167.

[8] Y. Lu, L. Wang, K. Preuß, M. Qiao, M. M. Titirici, J. Varcoe, Q. Cai, *J. Power Sources* **2017**, *372*, 82–90.

[9] H. Ren, Y. Wang, Y. Yang, X. Tang, Y. Peng, H. Peng, L. Xiao, J. Lu, H. D. Abruña, L. Zhuang, *ACS Catal.* **2017**, *7*, 6485–6492.

[10] Y. J. Sa, D.-J. Seo, J. Woo, J. T. Lim, J. Y. Cheon, S. Y. Yang, J. M. Lee, D. Kang, T. J. Shin, H. S. Shin, et al., *J. Am. Chem. Soc.* **2016**, *138*, 15046–15056.

[11] H. T. Chung, D. A. Cullen, D. Higgins, B. T. Sneed, E. F. Holby, K. L. More, P. Zelenay, *Science (80-.).* **2017**, *357*, 479–484.

[12] J. Wang, Z. Huang, W. Liu, C. Chang, H. Tang, Z. Li, W. Chen, C. Jia, T. Yao, S. Wei, et al., *J. Am. Chem. Soc.* **2017**, *139*, 17281–17284.

[13] L. Xin, Z. Zhang, Z. Wang, J. Qi, W. Li, *Front. Chem.* **2013**, *1*, 16.

[14] P. I. A. Serov, **2016**.

[15] O.-H. Kim, Y.-H. Cho, D. Y. Chung, M. J. Kim, J. M. Yoo, J. E. Park, H. Choe, Y.-E. Sung, *Sci. Rep.* **2015**, *5*, 8376.

[16] S. K. Singh, V. Kashyap, N. Manna, S. N. Bhange, R. Soni, R. Boukherroub, S. Szunerits, S. Kurungot, *ACS Catal.* **2017**, *7*, 6700–6710.

[17] T. J. Omasta, A. M. Park, J. M. LaManna, Y. Zhang, X. Peng, L. Wang, D. L. Jacobson, J. R. Varcoe, D. S. Hussey, B. S. Pivovar, et al., *Energy Environ. Sci.* **2018**, *11*, 551–558.

[18] T. J. Omasta, Y. Zhang, A. M. Park, X. Peng, B. Pivovar, J. R. Varcoe, W. E. Mustain, *J. Electrochem. Soc.* **2018**, *165*, F710–F717.
